# Behavior Training Reverses Asymmetry in Hippocampal Transcriptome of the *Ca_v_3.2* Knockout Mice

**DOI:** 10.1371/journal.pone.0118832

**Published:** 2015-03-13

**Authors:** Ni-Chun Chung, Ying-Hsueh Huang, Chuan-Hsiung Chang, James C. Liao, Chih-Hsien Yang, Chien-Chang Chen, Ingrid Y. Liu

**Affiliations:** 1 Department of Molecular Biology and Human Genetics, Tzu Chi University, Hualien, Taiwan; 2 Institute of Biomedical Informatics, National Yang-Ming University, Taipei, Taiwan; 3 Department of Chemical and Biomolecular Engineering, University of California Los Angeles, Los Angeles, California, United States of America; 4 Institute of Biomedical Sciences, Academia Sinica, Taipei, Taiwan; 5 Institute of Medical Sciences, Tzu Chi University, Hualien, Taiwan; Western University of Health Sciences, UNITED STATES

## Abstract

Homozygous *Ca_v_3.2* knockout mice, which are defective in the pore-forming subunit of a low voltage activated T-type calcium channel, have been documented to show impaired maintenance of late-phase long-term potentiation (L-LTP) and defective retrieval of context-associated fear memory. To investigate the role of *Ca_v_3.2* in global gene expression, we performed a microarray transcriptome study on the hippocampi of the *Ca_v_3.2^-/-^* mice and their wild-type littermates, either naïve (untrained) or trace fear conditioned. We found a significant left-right asymmetric effect on the hippocampal transcriptome caused by the *Ca_v_3.2* knockout. Between the naive *Ca_v_3.2^-/-^* and the naive wild-type mice, 3522 differentially expressed genes (DEGs) were found in the left hippocampus, but only 4 DEGs were found in the right hippocampus. Remarkably, the effect of *Ca_v_3.2* knockout was partially reversed by trace fear conditioning. The number of DEGs in the left hippocampus was reduced to 6 in the *Ca_v_3.2* knockout mice after trace fear conditioning, compared with the wild-type naïve mice. To our knowledge, these results demonstrate for the first time the asymmetric effects of the *Ca_v_3.2* and its partial reversal by behavior training on the hippocampal transcriptome.

## Introduction

The *Ca*_*v*_ *3*.*2* (*Cacna1h*) gene encodes the pore-forming subunit of the low voltage activated *α*_*1H*_ T-type calcium channel [1], which is highly expressed in the hippocampal formation and has been implicated in a variety of physiological processes [2–4]. The neuronal *α*_*1H*_ T-type calcium channel can generate low-threshold spikes that lead to burst firing and oscillatory behavior [5–7]. Alterations in these oscillations have been found in a wide range of neurological disorders including schizophrenia [8,9], Parkinson’s disease [10,11], and chronic atypical absence seizures [12]. Mutations in the human *Ca*_*v*_ *3*.*2* homologue, *Cacna1H*, that enhance channel activity have been associated with childhood absence epilepsy [13–17], whereas mutations that disrupt channel function have been associated with autism spectrum disorders [18]. These findings suggest a critical role of the *Ca*_*v*_ *3*.*2* in the central nervous system (CNS).

The *Ca*_*v*_ *3*.*2* gene also plays an important role in memory formation. We have reported that the *Ca*_*v*_ *3*.*2* ^*-/-*^ knockout mice (*Cacna1h* ^*tm1Kcam*^) are impaired in maintenance of late-phase long-term potentiation (L-LTP) and in retrieval of hippocampal-dependent contextual memory, while their locomotion function and ability of learning spatial cues are normal [19]. To further probe the effects of the *Ca*_*v*_ *3*.*2* gene loss on global gene expression patterns, here we performed a microarray analysis to establish the hippocampal gene expression profiles of the naïve and trace fear conditioned *Ca*_*v*_ *3*.*2* ^*-/-*^ mice and their WT littermates. In particular, we wished to determine the interaction between the *Ca*_*v*_ *3*.*2* and behavior training such as trace fear conditioning (TFC). Since gene expression patterns in the left and right hippocampi have been shown to differ in developing rats [20] and in rats subjected to hidden platform test of Morris water maze [21], we dissected bilateral hippocampi separately for microarray analysis.

We found that the *Ca*_*v*_ *3*.*2* ^*-/-*^ knockout exhibited a significant left-right asymmetric effect on hippocampal gene expression. This effect confirmed known asymmetric functions of left and right hippocampi [22] at the molecular level. Remarkably, behavior training using the TFC protocol largely reversed the effect of the *Ca*_*v*_ *3*.*2* gene knockout. This finding suggests that behavior training may partially and at least transiently compensate effects of the gene knockout. To our knowledge, this is the first report of behavior training reversing the effect of a gene knockout on global gene expression pattern in the hippocampi.

## Materials and Methods

### Ethics statement

All protocols used in this study have been reviewed and approved by Institutional Animal Care and Use Committee of Tzu Chi University (Approval ID 95130) and complied with “Guidelines for Ethical Treatment of Animals” provided by Taiwan’s Ministry of Science and Technology.

### Animals

C57BL/6J WT, male mice, originally provided from Taiwan’s Ministry of Science and Technology, were purchased and maintained undisturbed in the laboratory animal center of Tzu Chi University until the behavioral tasks were performed. The *Ca*_*v*_ *3*.*2* ^*-/-*^ mice were generated and genotyped as described previously [23]. The heterozygous (*Ca*_*v*_ *3*.*2* ^*+/-*^) mice have been bred to C57/BL6 background for 7 generations. Amplification of exon 6 was performed using PCR to genotype the individual mice before behavioral training. Animals were housed individually in plastic and metal cages with *ad libitum* access to food and water under a constant 12hr light/dark cycle. All experiments were performed in a blinded manner. Hippocampal RNA extraction from three pairs of hippocampi of three mice was used for each group. Twenty-four mice in 8 groups (Wildtype Naïve Left-hippocampus: WNL; Wildtype Naïve Right-hippocampus: WNR; Wtildtype Training Left-hippocampus: WTL; Wildtype Training Right-hippocampus: WTR; Knockout Naïve Left-hippocampus: KNL; Knockout Naïve Right-hippocampus: KNR; Knockout Training Left-hippocampus: KTL; and Knockout Training Right-hippocampus: KTR), were used for one microarray analysis and two replicates of microarray analysis were performed. A total of 16 microarrays were used in this study.

### Trace fear conditioning

The mice were placed into the conditioning chamber for 15 minutes per day for three days prior to training for them to adapt to the new environment. On the training day (the 4^th^ day), the mice received three TFC trials that began with a 20 sec tone (CS), followed by a 10 sec trace period before the animal received a 1 sec foot shock (2mA, US). The inter-training interval is 1 min. The mice were then placed for 6 minutes into the same conditioning chamber for the contextual test 24 hours later (on the 5^th^ day). Mice were decapitated after contextual testing for further hippocampal dissection and RNA extraction.

### RNA preparation

Three left and three right hippocampi from WT and KO respectively were dissected, pooled together and homogenized in 1 ml Trizol (TRIzol. Invitrogen, CA, USA) by micro centrifuge pestle (PP Micro Centrifuge Sample Pestle, Foremost Product Intl., CA, USA). After chilling on ice for 5 min, samples were added with 0.2 ml chloroform (Chloroform, J.T. Baker, NJ, USA) and then vortexed for 25 sec and stayed on ice for 10 min. After centrifuged at 13,000 rpm, 4°C for 10 min, 500 μl supernatant of each sample were transferred to clean tubes, and 0.5 ml isopropanol (2-propanol, J.T. Baker, NJ, USA) was added with properly vortex. Tubes were centrifuged at 13,000 rpm, 4°C for 20 min, supernatant was poured off and RNA pellet was collected. Then 1 ml 75% ethanol (Ethanol absolute, Sigma-Aldirich, Germany) was added to each tube for dehydration. Supernatant was removed after centrifugation for 10 min, and pellet was dissolved in 60 to 80μl ddH_2_O. Quality of extracted RNA was checked by reading the ratio of absorbance at 260 nm versus 280 nm by NanoDrop ND-1000 (NanoDrop, DE, USA). Samples with the 260/280 ratio of 1.8–2.2 were accepted for further microarray analysis. RNA structural intactness and DNA contamination were checked by Agilent RNA 6000 Nano Assay (Agilent, HOA, Phalanx) and RNA agarose gel electrophoresis.

### Microarray hybridization

Microarray hybridization was commissioned to Phalanx Biotech Group, Taiwan. RNA samples were hybridized to Mouse Whole Genome OneArray TM (MOAV1.1) with 29,922 mouse genome probes and 1880 experimental control probes, which were an abridged version of the MEEBO probe set (http://genome.ucsc.edu). The 70-mer aminoallyl-aRNA [24] targets were amplified using MessageAmp aRNA kit (Amnion) and labeled with the NHS-Cy5 (GE). The labeled targets were hybridized at 50°C for 16 hr to the gene chip. After washing 3 steps (42°C, 5 min twice, and 25°C, 5 min), the array was exposed to a phosphorimaging cassette and then scanned with Axon 4000B Scanner (Molecular Devices) as well as analyzed with Genepix software (Molecular Devices). Two technical replicates were averaged (reproducibility >0.95), and the averaged intensities were normalized. The normalized data was computed with log_2_ after comparing to each other. The entire raw dataset were deposited in the Gene Expression Omnibus (GSE30846).

### Microarray data analysis

**Fig 1:**
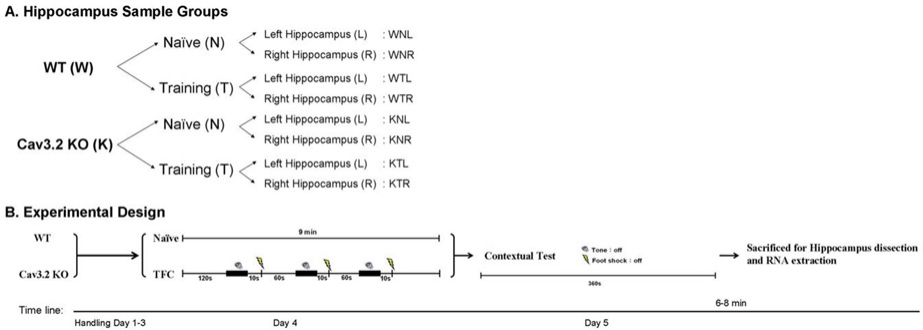
The overall experimental design. (A) Hippocampal sample groups. We used microarrays to probe the transcriptome of naïve (N) and trained (T) groups for both wild-type (W) and the *Ca*_*v*_ *3*.*2* homozygous knockout (K) mice. In each group, we separated left (L) and right (R) hippocampi samples. A total of eight groups of samples were analyzed: WNL, WNR, WTL, WTR, KNL, KNR, KTL, and KTR. (B) Experimental procedures: Mice were handled in conditioning chambers for three days (Day 1–3), and then received trace fear conditioning individually (Day 4), tested with contextual memory 24 hours later (Day 5) in the same conditioning chamber. Immediately after memory testing, left (L) and right (R) hippocampi from three W and three K animals were dissected, pooled together to extract total RNA for microarray hybridization.

Bioconductor (www.bioconductor.org) and R (www.r-project.org) were used as computational tools for microarray analysis. Raw data were thresholded with a floor of 100 and normalized using the quantile normalization method. To identify differentially expressed genes (DEGs), the Significance Analysis of Microarrays (SAM) algorithm [25] and fold change (FC) were used for each of eight microarray comparisons (WNL vs KNL, WNR vs KNR, WTL vs KTL, WTR vs KTR, WNL vs WTL, KNL vs KTL, WNR vs WTR, KNR vs KTR, Fig. 1A). A gene was considered significantly *differentially expressed* if a false discovery rate (q) is less than 0.01 and FC is greater than 2. The DEGs identified in at least one of comparisons were clustered using a hierarchical clustering method. To gain insight into the biological processes involved, each cluster was then analyzed by DAVID functional annotation tool [26] to identify *enriched KEGG pathways*. In order to demonstrate the repeatability of the duplicated microarray raw datasets, we perform correlational analysis. S1 Table lists the designated groups of raw datasets. S1 and S2 Figs. and show the scatter plots of the raw datasets of the control and the training groups, respectively. Correlation analysis shows that for all groups R≥ 0.99, indicating that the consistency of the duplicate raw datasets is high.

### Real time RT-PCR

For each selected gene, hippocampal RNA was extracted bilaterally from three mice for each group, and the real time RT-PCR was triplicated. Hippocampal RNA isolated bilaterally from each group of mice was reverse transcribed to cDNA using the High-Capacity cDNA Reverse Transcription Kits (Applied Biosystems, CA, USA). Two micrograms of RNA from each template sample was incubated with 2 μl of 10X RT buffer, 0.8 μl 25X dNTP mix (100 mM), 2 μl 10X RT random primers, and 1 μl MultiScribe^TM^ reverse transcriptase in a 20μl aqueous solution. The mixtures were then incubated at 25°C for 10 min, 37°C for 120 min, and 85°C for 5 sec. The reactions were then stopped and the tubes were maintained at 4°C.

Primers that amplified the selected genes were designed using Primer 3 (http://bioinfo.ut.ee/primer3) [27,28] based on the mRNA sequences listed in NCBI. All primers were checked by agarose gel electrophoresis following regular PCR before being used for Real-Time Quantitative PCR. The product sizes ranged from 100 to 150 bp, the T_M_ value was 57 to 59°C, and the GC content was 50 to 80%. We considered the ideal characteristics to be a 100-bp product size, 58°C T_M_ value, and 50% GC content would be the first choice.

Two microliters of cDNA from each template sample were added to 10μl SYBR Green PCR Master Mix (Applied Biosystems), followed by 2μl of 5μM forward and reverse primers and ddH_2_O to a final volume of 20μl. The mixed solutions were placed in a ABI 7300 Real-Time PCR system (Applied Biosystems) and subjected to 5 steps: (a) 50°C for 2min (b) 94°C for 4min (c) 94°C for 1min (d) 58°C for 1min and (e) 72°C for 1min. Steps (c) to (e) were repeated for 40 cycles.

The qRT-PCR results were analysed using the ABI 7300 Real-Time PCR RQ study software (Applied Biosystems, CA, USA), and are displayed as Ct values acquired exponential phase of amplification. The relative quantitative threshold cycle method (ΔΔ*Ct*) was used to determine target gene expression relative to the internal control gene, β-actin. The level of target gene expression was calculated as 2^-ΔΔ*Ct*^, where ΔΔCt = [Ct β-actin (naïve group)—Ct selected gene (naïve group)]—[Ct β-actin (training group)—Ct selected gene (training group)]. Listed expression level for each gene in S2 Table was the mean value of triplicate results.

## Results

### Overall experimental design

**Fig 2:**
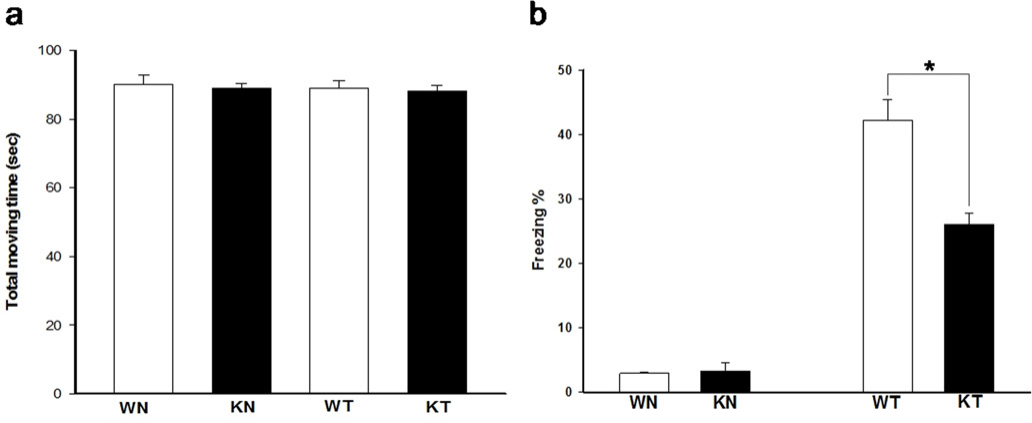
Behavioral analysis. (a) Locomotor activity for all groups measured during the first 120 seconds of the training protocol showed that the *Ca*_*v*_ *3*.*2* knockout mice are as normal as their wild-type littermates. (b) The *Ca*_*v*_ *3*.*2* knockout mice were impaired in retrieval of contextual memory tested 24 hours post the TFC training protocol. (WN: wild-type naïve group; KN: knockout naïve group; WT: wild-type training group; KT: knockout training group; * p<.05)

To investigate the role of the *Ca*_*v*_ *3*.*2* and the effect of TFC on the *Ca*_*v*_ *3*.*2* ^*-/-*^ mice, we used microarrays to probe the transcriptome of naïve (N) and trained (T) groups for both wild-type (W) and the *Ca*_*v*_ *3*.*2* homozygous knockout (K) mice. Wild-type C57BL/6J male mice were maintained undisturbed in the laboratory animal center until the behavioral tasks were performed. The *Ca*_*v*_ *3*.*2* ^*-/-*^ mice were generated and genotyped as described previously [23]. Mice were trace fear conditioned individually and then tested with contextual memory 24 hours later in the same conditioning chamber. Immediately after memory testing, left (L) and right (R) hippocampi from three W and three knockout animals were dissected, pooled together to extract RNA. The RNA samples were amplified, reverse-transcribed to cDNA and labeled, and then hybridized to Mouse Whole Genome OneArray TM (MOAV1.1) with 29,922 mouse genome probes and 1880 experimental control probes. A total of eight groups of samples were analyzed: WNL, WNR, WTL, WTR, KNL, KNR, KTL, and KTR (Fig. 1A). The overall experimental design was illustrated in Fig. 1B. In order to reconfirm the phenotype of the *Ca*_*v*_ *3*.*2* knockout mice, we analyzed their behavioral data after TFC. Fig. 2A showed that the *Ca*_*v*_ *3*.*2* knockout mice demonstrated normal locomotor activity and Fig. 2B showed that their contextual memory retrieval was impaired as compared with their wild-type littermates. The deficiency in retrieval of contextual memory is consistent with our previous study [19], and demonstrates the robustness of the effect.

**Fig 3:**
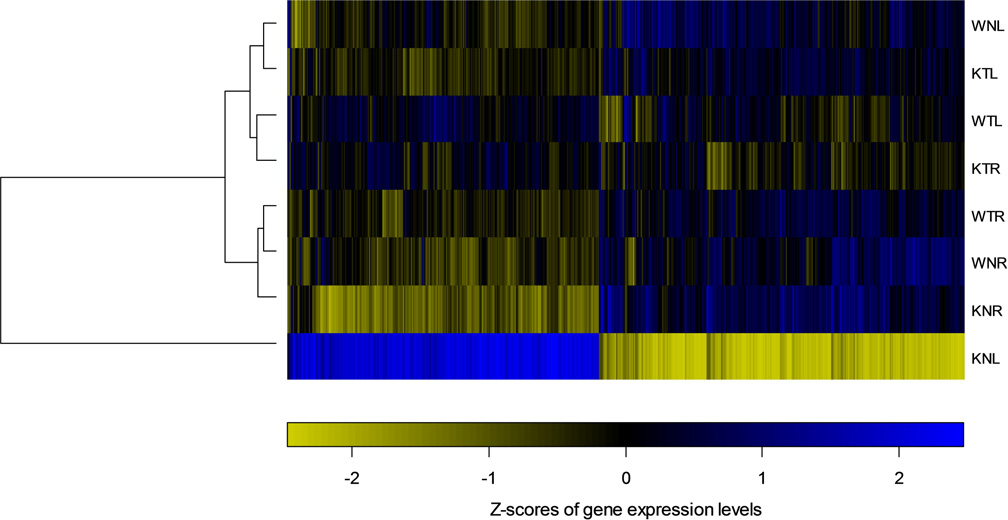
The heatmap representing the relative expression level of each DEG over eight sample groups. In the heatmap, color indicates the Z-score of expression relative to the mean over eight conditions. Eight conditions were clustered using hierarchical clustering.

Fig. 3 shows the heatmap of the DEG relative expression levels in the eight sample groups. To determine the effects of the *Ca*_*v*_ *3*.*2* knockout, we compared K and W samples in both N and T groups. In each group, we separated L and R hippocampi samples. To determine the effect of TFC, we compared T and N in both W and K groups, and also separated the samples into L and R hippocampi. To determine the effect of lateralization, we compared L and R in both W and K under T and N conditions (Table 1).

**Table 1:** Numbers of differentially expressed genes (DEGs) in each microarray comparison .

Treatments	Group# (Treatment vs Control)			Up-regulated	Down-regulated	Total
KO (K) vs WT (W)	Naïve	1	KNL vs WNL	1302	2220	3522
		2	KNR vs WNR	0	4	4
	Training	3a	KTL vs WTL	0	5	5
		4	KTR vs WTR	1	16	17
KO Training (KT) vs WT Naïve (WN)	Left	3b	KTL vs WNL	0	6	6
Left (L) vs Right (R)	Naive	5	WNL vs WNR	1	0	1
		6	KNL vs KNR	2233	2571	4804
	Training	7	WTL vs WTR	16	38	54
		8	KTL vs KTR	60	19	79
Training (T) vs Naïve (N)	WT	9	WTL vs WNL	0	3	3
		10	WTR vs WNR	6	0	6
	KO	11	KTL vs KNL	2132	1456	3588
		12	KTR vs KNR	217	241	458

### The *Ca*_*v*_ *3*.*2* knockout exhibited an asymmetric effect on hippocampal transcriptome

In naïve animals, we found that homozygous deletion of the *Ca*_*v*_ *3*.*2* exhibited a marked impact on the transcriptome profiles. Interestingly, transcription changes caused by the *Ca*_*v*_ *3*.*2* knockout were almost exclusively found in the left hippocampus (Table 1, #1), and only minor differences were found in the right hippocampus (Table 1, #2). Among the 29,922 probe sets, 3522 genes were differentially transcribed in the KNL vs WNL comparison, with 1302 genes up-regulated and 2220 down-regulated by loss of the *Ca*_*v*_ *3*.*2* gene (Table 1, #1). To gain greater biological insight, the DEGs of KNL vs WNL were then analyzed by using the Database for Annotation, Visualization and Integrated Discovery (DAVID) and the Kyoto Encyclopedia of Genes and Genomes (KEGG, www.genome.jp/kegg) to identify pathways involved [26]. The enriched pathways with 10 or more DEGs in KNL vs WNL comparison are listed in S3 Table. In particular, the MAPK signaling pathway, which has been known to be involved in contextual fear memory [29,30], has 45 up-regulated and 28 down-regulated genes in KNL compared with WNL. The long-term potentiation pathway, which is correlated with impaired hippocampal L-LTP in the *Ca*_*v*_ *3*.*2* ^*-/-*^ strain [19], has 14 up-regulated genes in the KNL compared to the WNL group. Up-regulated genes are also enriched in the neurotrophin signaling pathway, the citric acid cycle (TCA cycle), and pathways associated with neurodegenerative diseases such as Alzheimer’s, Huntington’s, and Parkinson’s diseases (http://www.genome.jp/kegg/pathway).

In contrast, the gene expression pattern in the right hippocampus was largely unchanged between the knockout mice with the wild-type mice (KNR vs WNR, Table 1, #2). Only 4 down-regulated genes were detected from the naïve, right hippocampi. The 4 DEGs in the KNR vs WNR group are listed in Table 2. One of them, Ube2i, is involved in the ubiquitin-mediated proteolysis pathway. To determine if the asymmetric transcriptome pattern already existed in the wild-type mice, we compared the left and right hippocampi in the naïve wild-type mice and in the knockout mice. The wild-type did not show an asymmetric transcriptome pattern (WNL vs WNR Table 1, #5), whereas the knockout mice exhibited significant lateral differences (KNL vs KNR, Table 1, #6), as expected from the above results. These results demonstrate that *Ca*_*v*_ *3*.*2* ^*-/-*^ exhibits a lateral asymmetric effect on the hippocampal transcriptome.

**Table 2:** Differentially expressed genes in KNR vs WNR group.

Gene Symbol	Description	Gene ID	q value(%)	Fold Change	
Lemd2	LEM domain containing 2	224640	0	0.20+1.08	Down
Ube2i	ubiquitin-conjugating enzyme E2I	22196	0	0.24+0.69	Down
Lce1c	late cornified envelope 1C	73719	0	0.26	Down
Panx3	pannexin 3	208098	0	0.25	Down

### Trace fear conditioning significantly reversed the asymmetrical gene expression pattern caused by the *Ca*_*v*_ *3*.*2* knockout

To determine the effect of TFC, we first compared wild-type mice with or without TFC, and found that TFC did not alter the transcriptome of either left or right hippocampus in the wild-type mice (WTL vs WNL, and WTR vs WNR, Table 1, #9, 10), based on the 2-fold cut-off. Only minor changes were found: 3 down-regulated genes were identified in the left (Table 3) and 6 up-regulated genes were found in the right (Table 4) hippocampi of the wild-type mice after training. However, TFC altered the transcriptome patterns of the knockout mice dramatically, particularly in the left hippocampus. As seen in the comparison between KTL vs KNL (Table 1, #11), TFC had a significant impact on the left hippocampus but a relatively small effect on the right hippocampus (KTR vs KNR, Table 1, #12) in the *Ca*_*v*_ *3*.*2* ^*-/-*^ knockout mice. These results suggest that significant interactions exist between TFC and the *Ca*_*v*_ *3*.*2* ^*-/-*^ in the left hippocampus.

**Table 3:** Differentially expressed genes in WTL vs WNL group.

Gene Symbol	Description	Gene ID	q value(%)	Fold Change	
Ss18	synovial sarcoma translocation, Chromosome 18	268996	0	0.33	Down
Mark3	MAP/microtubule affinity-regulating kinase 3	17169	0	0.33	Down
Cyb561d1	cytochrome b-561 domain containing 1	72023	0	0.42	Down

**Table 4:** Differentially expressed genes in WTR vs WNR group.

Gene Symbol	Description	Gene ID	q value(%)	Fold Change	
Ddx19b	DEAD (Asp-Glu-Ala-Asp) box polypeptide 19b	234733	0	3.29	Up
Mrps6	mitochondrial ribosomal protein S6	121022	0	7.04	Up
2310061I04Rik	RIKEN cDNA 2310061I04 gene	69662	0	2.66	Up
Mt3	metallothionein 3	17751	0	2.39	Up
Tbca	tubulin cofactor A	21371		2.41	Up
Lst1	leukocyte specific transcript 1	16988		2.44	Up

Interestingly, the profound effect of the *Ca*_*v*_ *3*.*2* knockout on the left hippocampus in naïve mice was significantly reduced after training. The comparison between KTL and WTL (Table 1, #3a) showed only minor differences. We found only 5 genes differentially expressed by knockout (Table 5). For example, the MAPK signaling pathway genes that were upregulated in the *Ca*_*v*_ *3*.*2* knockout mice were no longer upregulated after training. In the R samples, the difference between K and W was slightly increased in the trained group (Table 1, #4) compared to the naïve group (Table 1, #2). However, the difference is relatively small, only 1 gene was up-regulated and 16 genes were down-regulated (Table 6). Most interestingly, by comparing KTL with WNL (Table 1, #3b), we also saw little difference: only 6 DEGs were found. These results suggest that TFC largely reverses the effect of the *Ca*_*v*_ *3*.*2* gene knockout at least at the transcriptome level within the experimental time frame.

**Table 5:** Differentially expressed genes in KTL vs WTL group.

Gene Symbol	Description	Gene ID	q value(%)	Fold Change	
Mrps6	mitochondrial ribosomal protein S6	121022	0	0.04	Down
Ube2i	ubiquitin-conjugating enzyme E2I	22196	0	0.23+0.82	Down
Kcne2	potassium voltage-gated channel, Isk-related subfamily, gene 2	246133	0	0.29	Down
Ptprg	protein tyrosine phosphatase, receptor type, G	19270	0	1.91+0.28	Down
Lemd2	LEM domain containing 2	224640	0	0.31+1.20	Down

**Table 6:** Differentially expressed genes in KTR vs WTR groups.

Gene Symbol	Description	Gene ID	q value(%)	Fold Change	
Erdr1	erythroid differentiation regulator 1	170942	0	2.72	Up
Lemd2	LEM domain containing 2	224640	0	0.33	Down
Ndufb10	NADH dehydrogenase (ubiquinone) 1 beta subcomplex, 10	68342	0	0.27	Down
Ppp1r3c	protein phosphatase 1, regulatory (inhibitor) subunit 3C	53412	0	0.32	Down
Pgbd5	piggyBac transposable element derived 5	209966	0	0.33	Down
Ddx19b	DEAD (Asp-Glu-Ala-Asp) box polypeptide 19b	234733	0	0.31	Down
Sdccag1	serologically defined colon cancer antigen 1	66244	0	0.35	Down
Clcn7	chloride channel 7	26373	0	0.28	Down
Rraga	Ras-related GTP binding A	68441	0	0.36	Down
2610002M06Rik	RIKEN cDNA 2610002M06 gene	67028	0	0.35	Down
Ranbp2	RAN binding protein 2	19386	0	0.37	Down
Eme2	essential meiotic endonuclease 1 homolog 2 (S. pombe)	193838	0	0.42	Down
Cldn12	claudin 12	64945	0	0.40	Down
Ogn	osteoglycin	18295	0	0.42	Down
Spink8	serine peptidase inhibitor, Kazal type 8	78709	0	0.40	Down
Gabrb2	gamma-aminobutyric acid (GABA) A receptor, subunit beta 2	14401	0	0.46	Down
2610019E17Rik	RIKEN cDNA 2610019E17 gene	75614	0	0.47	Down

Since TFC had relatively small effects on wild-type left or right hippocampi (Table 1, #9,10), and since wild-type mice did not show asymmetric transcriptome (Table 1, #5), it was not surprising that TFC showed no asymmetric effect on wild-type mice (WTL vs WTR, Table 1, #7). In addition, comparison of KTL vs KTR (Table 1, #8) also showed relative minor differences. This was because TFC largely reversed the effect of the *Ca*_*v*_ *3*.*2* gene knockout on the left hippocampus, and TFC and *Ca*_*v*_ *3*.*2* gene knockout had little impact on the right hippocampus, according to the data described above.

### Principal component analysis

**Fig 4:**
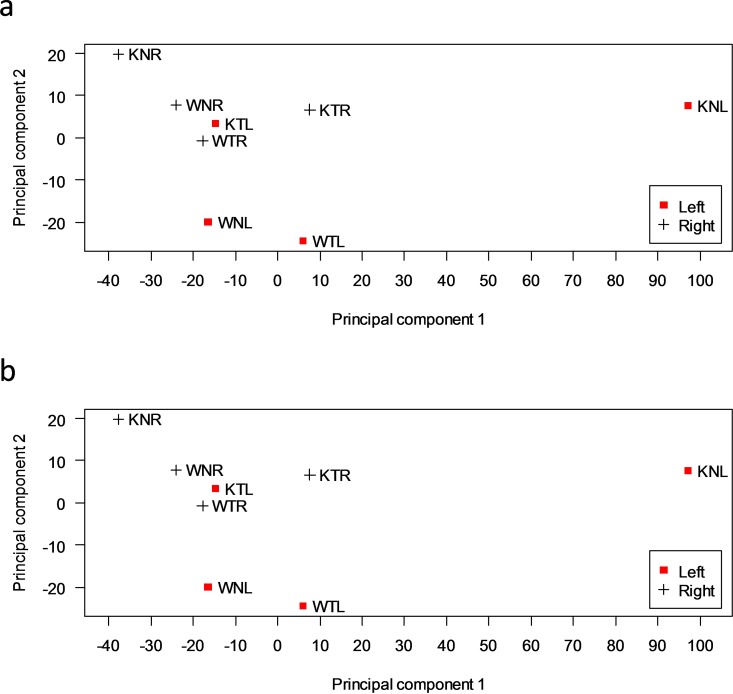
Principal component analysis (PCA) is used to cluster the conditions with gene expression levels for (a) all genes, (b) 5282 genes that are differentially expressed in at least one of comparisons listed in Table 1. Each point corresponds to the gene expression pattern generated by an experimental group projected onto the two-dimensional principal component space.

To summarize the effects of *Ca*_*v*_ *3*.*2* gene knockout and TFC, on the left and right hippocampal transcriptome profiles, we analyzed the data by using Principal Component Analysis (PCA), and then plotted the first two Principal Components. The PCA plots allow visualizing the distance between different data sets in the dimensions that exhibit the major variations. We used all genes (Fig. 4a) and 5282 genes which were differentially expressed in at least one of comparisons listed in Table 1 (Fig. 4b) for each comparison group to plot the PCA. These two analyses gave almost identical results. Fig. 4 shows that KNL is farthest away from the rest of the data sets in the first principal component (PC1), indicating that the *Ca*_*v*_ *3*.*2* gene knockout exhibited the largest effect on the transcriptome pattern. The rest of the data sets more or less clustered together, relative to KNL. By comparing KTL with KNL, we noted that after training (KTL), the trancriptome pattern of KNL returned to the same cluster as the WTL and WNL in PC1. This result confirmed that TFC largely reversed the effect of the *Ca*_*v*_ *3*.*2* gene knockout. The second principal component (PC2) mainly separated between L and R samples in the wild-type (Fig. 4). Even though training reversed the effect of the *Ca*_*v*_ *3*.*2* knockout in PC1, KTL transcriptome pattern did not return to the cluster of WTL and WNL in PC2. Rather, KTL was closer to the R samples in the PC2 dimension. This result indicates that behavioral training did not completely reverse the knockout effect, and the residual difference made the KTL pattern resemble the R samples.

### Validation by quantitative reverse transcription polymerase chain reaction

To confirm the microarray data, we used quantitative reverse transcription polymerase chain reaction (qRT-PCR) to validate expression differences. The ΔΔ CT method was used to calculate the fold differences for each of the genes and qRT-PCR validation results were summarized in S2 Table. For each gene, the qRT-PCR result listed in S2 Table was the mean value of triplicate results. A total of 101 genes were selected from the DEGs in the comparison between KNL and WNL groups, including genes representing the MAPK, ubiquitin-mediated proteolysis, and LTP pathways, which were enriched in the KNL vs WNL comparison. Consistent with the array data, 88 genes (87.1%) demonstrated differentially expressed patterns in KNL compared with WNL, based on the 2-fold cut-off. Expression levels of these genes returned to WT levels after trace fear conditioning (S2 Table). Although there were 3522 DEGs in the left hippocampi between the naïve K and W groups (KNL vs WNL, Table 1, #2), the number of DEGs was reduced to 5 after trace fear conditioning (KTL vs WTL, Table 1, #3a). Kcne2, Ptprg, and Lemd2 were selected out of the 5 genes to validate this phenomenon. Consistent with the array data, they were significantly down regulated in the left hippocampi of the KO after training and testing (KTL) compared with the WTL sample (S2 Table).

## Discussion

In this work, we demonstrated that the *Ca*_*v*_ *3*.*2* ^*-/-*^ mice exhibited a distinct hippocampal gene expression pattern compared with that of their WT littermates. Interestingly, the effect of the *Ca*_*v*_ *3*.*2* ^*-/-*^ on the hippocampal transcriptome was asymmetrical: *Ca*_*v*_ *3*.*2* ^*-/-*^ had a significant impact on the left hippocampus but not on the right. This asymmetric effect is quite intriguing, as no other similar effect has been reported to our knowledge after extensive literature search. To validate the microarray results, we used qRT-PCR to re-examine 101 genes from the DEGs from the comparison between KNL and WNL group. Indeed, 87% of them showed a difference between KNL and WNL.

Asymmetry in brain [31–33], and in particular in hippocampal formation [34–37] has been well recognized, but the molecular mechanisms underlying the asymmetry remain mostly unknown [31,38]. Asymmetry in neuronal architecture and connectivity have been reported [32,34], and asymmetry in molecular distributions has also been reported for hippocampal glutamate receptor expression [34] and NMDA Receptor 2 Subunits [37]. In human, the left hippocampus is involved in verbal memory [39] and the right with spatial memory [40]. Impaired lateralization of the hippocampus and associated limbic structures has been reported for patients affected by autism and schizophrenia [41]. In rodents and rats, electro-physiological recording have shown that spatial processing is lateralized in the right hemisphere, while motor control and time analysis in the left [22].

Our results demonstrate that loss of the *Ca*_*v*_ *3*.*2* amplifies the asymmetry already exists in hippocampi. The *Ca*_*v*_ *3*.*2* gene itself was not differentially transcribed between left and right hippocampi in the wild-type mice, based on the 2-fold cut-off of our trancriptome data. Since the effect of the *Ca*_*v*_ *3*.*2* knockout was mainly seen in the left hippocampus, it is possible that the *Ca*_*v*_ *3*.*2* protein itself is not functional in the right hippocampus due to other regulatory mechanisms, or other calcium channels in the right hippocampus could compensate for the loss of *Ca*_*v*_ *3*.*2*.

This asymmetric effect may subsequently lead to better understanding of functional lateralization. Asymmetric gene expression patterns in the left and right hippocampi have been shown to differ in developing rats [20] and in rats subjected to the hidden platform test of Morris Water Maze [21]. Klur et al [21] further confirmed a functional association between lateralized transcriptional activity within the dorsal hippocampus and spatial memory in the rat. They showed that in mice after Morris Water Maze testing, 623 genes were differentially expressed in the right hippocampus, whereas only 74 were differentially expressed in the left hippocampus. Early experience of reward through maternal contact or its denial also affects laterality of protein expression in the developing rat hippocampus [42].

Our microarray analysis confirms Bianki’s (1981) findings [22] and explains the fact that the *Ca*_*v*_ *3*.*2* knockout strain exhibits intact spatial memory because the gene expression pattern in the right hippocampus does not alter much by loss of this gene, while temporal-associated memory (contextual trace fear memory) is impaired due to a pronounced change of the transcriptome in the left hippocampus.

There were 3522 genes differentially expressed in the WNL vs KNL group: 1302 genes were up-regulated and 2220 were down-regulated. On the other hand, the difference of gene expression patterns between WNR and KNR groups was much less. Only 4 down-regulated genes were identified from the naïve, right hippocampi. However, the significantly different numbers of expressed genes for the WNL vs KNL comparison was reduced after mice were trained with trace fear conditioning and tested with memory retrieval to context.

In the comparison between KNL and WNL, several enriched KEGG pathways are worth noting: 1) biochemical pathways such as splicesome, proteasome, oxidative phosphorylation, ribosome, citric acid cycle (TCA cycle), steroid biosynthesis, and ubiquitin mediated proteolysis; 2) pathways of neurodegenerative diseases such as Parkinson’s disease, Huntington’s disease, and Alzheimer’s disease; 3) Signal transduction pathways such as MAPK signaling and olfactory transduction. These biochemical pathways were also found by Klur et al [21] to be enriched after Morris water maze training. These pathways are expected to be necessary for maintenance of L-LTP and memory retrieval. *De novo* Protein synthesis has been known to be critical for stabilizing LTP [43] and memory retrieval [44], as well as protein degradation [45]. The TCA cycle is expected to generate ATP, NADH, and precursors to be used in numerous biochemical pathways. However, its importance in memory formation has not been studied. Our analysis provides a direction for future investigation in neuronal cell metabolism during processing of memory formation. Our analysis also implies the importance of the *Ca*_*v*_ *3*.*2* T-type calcium channel in pathways for three major neurodegenerative diseases and olfactory transduction in regards to memory retrieval. MAPK pathway has been known to be critical for LTP and retrieval of contextual fear memory [46–48]. Our results confirm these previous findings and indicate that the MAPK signaling may be dependent on calcium influx via the *Ca*_*v*_ *3*.*2* T-type calcium channel in retrieval of contextual memory.

After training, only 5 DEGs were identified for KTL vs WTL comparison (Table 1, 3a) including mitochondrial ribosomal protein S6 (Mrps6); ubiquitin-conjugating enzyme E2I (Ube2i); potassium voltage-gated channel, Isk-related subfamily, gene 2 (Kcne2); protein tyrosine phosphatase, receptor type, G (Ptprg); LEM domain containing 2 (Lemd2). Thus, the 5 DEGs may be linked to the severity of impaired phenotypes (defective in maintenance of L-LTP and in retrieval of contextual memory) of the *Ca*_*v*_ *3*.*2* knockout strain after TFC.

Perhaps the most unexpected and interesting result is that the effect of the *Cav3*.*2* knockout on the left hippocampal transcriptome is largely reversed by TFC. This is seen by the reduced number (only 6) of DEGs between KTL and WNL (Table 1, #3b) compared with the number of DEGs (3522) between KNL to WNL (Table 1, #1). Unfortunately, because measuring transcriptome profiles of additional controls (tone-alone, context alone, shock-alone.) became impractical, we could not conclusively demonstrate the effect of each step in the training protocol. We can only establish that training using the TFC protocol was responsible for the gene expression changes. There are many studies showing that large-scale gene expression patterns can be changed after behavioral training. Gene expression profiles in the hippocampus and amygdala have been found to be significantly different after fear conditioning [49]. In addition, gene expression profiling also changed and revealed a strong activation of immunity and inflammation responses in the brains of 10-month-old presenilin 1 and 2 conditional double knockout mice [50].

Our result indicates that the TFC protocol compensates for the *Ca*_*v*_ *3*.*2* knockout at least at the transcriptome level and within the experiment time frame. Previous studies have shown that calcium/calmodulin-dependent protein kinase IV (CaMKIV) [51] and L-type voltage-gated calcium channel [52] are involved in TFC. While the underlying mechanism remains unclear, it is possible that TFC activates other calcium channels, which at least partially and transiently compensate the deficit of the *Ca*_*v*_ *3*.*2*.

Calcium is known to be an important nuclear signal regulating a large number of genes [53–56] in all tissues and in brain specifically [55,57,58]. Nuclear calcium has been shown to be one of the most potent signals in gene expression in hippocampal neurons [55]. Nuclear calcium regulates hundreds of genes, as demonstrated by using cultured hippocampal neuron cell cultures and DNA microarray analysis [55]. In addition, calcium-activated potassium channels (BK channels) transduce signals from synapse to nucleus via calcium release to regulate gene expression [56]. Since both the *Ca*_*v*_ *3*.*2* knockout and TFC [51,52] affect calcium levels, it is possible that loss of the *Ca*_*v*_ *3*.*2* is compensated by calcium release induced by TFC and the gene expression pattern largely returns to the normal wild-type state. However, since both TFC and calcium-mediated transcription are highly complex, the detailed mechanism is still unknown. The situation is further complicated by the lateral asymmetry discussed above in both neuronal connectivity and molecular distribution.

Since transcription is the first step determining phenotypes, our result provides an example that behavioral disorders caused by a genetic mutation may be temporarily ameliorated by a behavioral training paradigm, and opens a possibility that behavior training may be used to reduce the severity of a symptom associated with a genetic defect. However, other factors may come to play in determining the proteome and metabolome, which are closer links to phenotypes.

This work also demonstrates the power of genome-scale analysis. Transcriptomic analysis reveals the global pattern of gene expression. Although overwhelmingly complex, the data can be analyzed systematically. In this case, PCA proves to be a useful tool to visualize the distance between different data set. Pathway analysis tools [26] yield detailed biochemical insight. Advanced methods such as Network Component Analysis [59,60] may generate further insight for future studies.

## Supporting Information

S1 Fig Correlational analysis for duplicate raw datasets of control groups. For all control groups (please see group designation in Table 1), R≥ 0.99, meaning that repeatability of duplicated microarray raw datasets is high.
(TIF)

S2 Fig Correlational analysis for duplicate raw datasets of training groups. For all training groups (please see group designation in S1 Table), R> 0.99, meaning that repeatability of duplicated microarray raw datasets is high.
(TIF)

S1 Table Designated groups of microarray raw datasets. (DOCX)

S2 Table Validation of selected DEGs by qRT-PCR. Expression patterns of selected DEGs highlighted in light grey are consistent with microarray data.
(DOCX)

S3 Table The enriched pathways with 10 or more DEGs in KNL vs WNL comparison. (DOCX)
