# Supplementary figures and images for: Behavior Training Reverses Asymmetry in Hippocampal Transcriptome of the Cav3.2 Knockout Mice
Source: PLoS One. 2015 Mar 13;10(3):e0118832. doi: 10.1371/journal.pone.0118832 (PMC4358833; doi:10.1371/journal.pone.0118832)

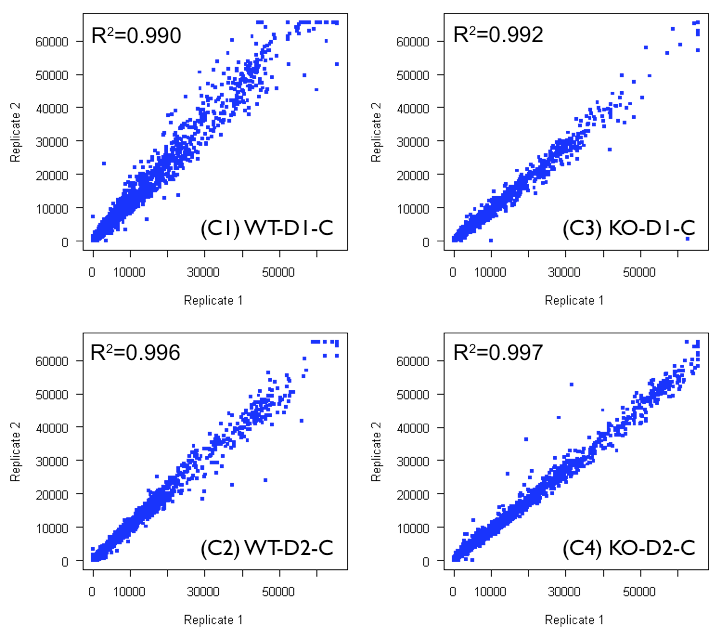

Supplement: S1 Fig — For all control groups (please see group designation in Table 1), R≥ 0.99, meaning that repeatability of duplicated microarray raw datasets is high. (TIF) [file pone.0118832.s001.tif]

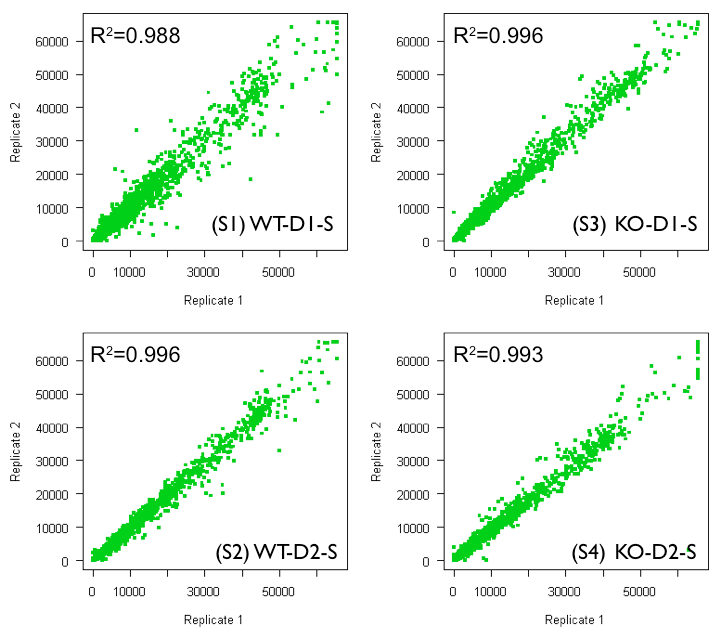

Supplement: S2 Fig — For all training groups (please see group designation in S1 Table), R> 0.99, meaning that repeatability of duplicated microarray raw datasets is high. (TIF) [file pone.0118832.s002.tif]
