# Supplementary material for: Behavior Training Reverses Asymmetry in Hippocampal Transcriptome of the Cav3.2 Knockout Mice
Source: PLoS One. 2015 Mar 13;10(3):e0118832. doi: 10.1371/journal.pone.0118832 (PMC4358833; doi:10.1371/journal.pone.0118832)
Supplement: S1 Table — (DOCX) [file pone.0118832.s003.docx]

**Table S1: Designated groups of microarray raw datasets.**

|  | WT | KO |
| --- | --- | --- |
| Data  Set #1 (D1) | C1_1 (C1 replicate #1): WT-D1-C-R1 (WNL_1)  C1_2 (C1 replicate #2): WT-D1-C-R2 (WNL_2)  S1_1 (S1 replicate #1): WT-D1-S-R1 (WTL_1)  S1_2 (S1 replicate #2): WT-D1-S-R2 (WTL_2) | C3_1 (C3 replicate #1): KO-D1-C-R1 (KNL_1)  C3_2 (C3 replicate #2): KO-D1-C-R2 (KNL_2)  S3_1 (S3 replicate #1): KO-D1-S-R1 (KTL_1)  S3_2 (S3 replicate #2): KO-D1-S-R2 (KTL_2) |
| Data  Set #2 (D2) | C2_1 (C2 replicate #1): WT-D2-C-R1 (WNR_1)  C2_2 (C2 replicate #2): WT-D2-C-R2 (WNR_2)  S2_1 (S2 replicate #1): WT-D2-S-R1 (WTR_1)  S2_2 (S2 replicate #2): WT-D2-S-R2 (WTR_2) | C4_1 (C4 replicate #1): KO-D2-C-R1 (KNR_1)  C4_2 (C4 replicate #2): KO-D2-C-R2 (KNR_2)  S4_1 (S4 replicate #1): KO-D2-S-R1 (KTR_1)  S4_2 (S4 replicate #2): KO-D2-S-R2 (KTR_2) |
