# Supplementary material for: Behavior Training Reverses Asymmetry in Hippocampal Transcriptome of the Cav3.2 Knockout Mice
Source: PLoS One. 2015 Mar 13;10(3):e0118832. doi: 10.1371/journal.pone.0118832 (PMC4358833; doi:10.1371/journal.pone.0118832)
Supplement: S2 Table — Expression patterns of selected DEGs highlighted in light grey are consistent with microarray data. (DOCX) [file pone.0118832.s004.docx]

**Table S2: Validation of selected DEGs by qRT-PCR. Expression patterns of selected DEGs highlighted in light grey are consistent with microarray data.**

| # | NAME | | Gene ID | Microarray fold-change | | | qRT-PCR fold-change | | |
| --- | --- | --- | --- | --- | --- | --- | --- | --- | --- |
| MAPK pathway | | |  | KNL/WNL | KTL/WNL | KTL/WTL | KNL/WNL | KTL/WNL | KTL/WTL |
| **1** | | **Fos** | 14281 | 0.41 | 0.51 | 1.09 | 0.52 | 0.67 | 1.12 |
| **2** | | **Atf2** | 11909 | 0.24+  1.88 | 0.91  +0.88 | 1.17+  0.77 | 1.97 | 0.95 | 0.92 |
| **3** | | **Bdnf** | 12064 | 0.28 | 0.90 | 1.19 | 0.32 | 1.20 | 1.13 |
| **4** | | **Cacna1c** | 12288 | 3.32+  2.24+  0.50 | 1.23+  1.21+  0.85 | 1.09+  0.97+  1.08 | 3.54 | 1.31 | 1.12 |
| **5** | | **Fgf12** | 14167 | 0.27 | 1.12 | 1.11 | 0.32 | 1.09 | 1.13 |
| **6** | | **Map2k1** | 26395 | 0.48 | 0.69 | 0.85 | 0.31 | 0.79 | 1.02 |
| **7** | | **Akt2** | 11652 | 0.40 | 1.02 | 1.19 | 0.35 | 1.13 | 2.12 |
| **8** | | **Nras** | 18176 | 0.25+  0.79 | 0.91+  1.54 | 1.06+  1.49 | 0.54 | 1.45 | 1.61 |
| **9** | | **Akt1** | 11651 | 0.26 | 0.83 | 0.94 | 0.31 | 1.02 | 1.05 |
| **10** | | **Tgfb2** | 21808 | 0.29 | 1.14 | 1.57 | 0.23 | 1.03 | 1.49 |
| **11** | | **Rela** | 19697 | 0.52 | 1.06 | 1.34 | 0.43 | 0.91 | 0.96 |
|  | | **NAME** |  | Microarray fold-change | | | qRT-PCR fold-change | | |
| **Ubiquitin mediated proteolysis pathway** | | |  | KNL/WNL | KTL/WNL | KTL/WTL | KNL/WNL | KTL/WNL | KTL/WTL |
| **12** | | **Traf6** | 22034 | 2.17+  0.39 | 1.25+  0.79 | 0.88+  0.82 | 3.12 | 1.01 | 1.29 |
| **13** | | **Cdc27** | 217232 | 0.44 | 1.01 | 1.04 | 0.51 | 1.02 | 1.12 |
| **14** | | **Map3k1** | 26401 | 0.42 | 0.89 | 1.08 | 0.35 | 1.09 | 2.21 |
| **15** | | **Ube2e3** | 22193 | 0.19+  0.44 | 0.99+  1.03 | 1.21+  1.01 | 0.25 | 1.17 | 1.25 |
| **16** | | **Mdm2** | 17246 | 0.39 | 0.91 | 1.02 | 0.41 | 1.20 | 1.19 |
| **17** | | **Ube3c** | 100763 | 0.36 | 0.86 | 1.17 | 0.29 | 1.09 | 1.32 |
| **18** | | **Vhl** | 22346 | 0.25 | 1.05 | 0.99 | 0.20 | 1.03 | 1.12 |
|  | | **NAME** |  | Microarray fold-change | | | qRT-PCR fold-change | | |
| **Long-term potentiation pathway** | | |  | KNL/WNL | KTL/WNL | KTL/WTL | KNL/WNL | KTL/WNL | KTL/WTL |
| **19** | | **Cacna1c** | 12288 | 3.32+  2.24+  0.50 | 1.23+  1.21+  0.85 | 1.09+  0.97+  1.08 | 3.54 | 1.31 | 1.12 |
| **20** | | **Gria1** | 14799 | 1.20+  1.14+  0.31 | 1.23+  1.04+  0.75 | 1.06+  0.97  0.87 | 1.25 | 1.18 | 1.21 |
| **21** | | **Calm1** | 12313 | 0.37 | 0.64 | 0.60 | 0.32 | 0.57 | 1.25 |
| **22** | | **Camk2g** | 12325 | 0.47 | 1.14 | 1.20 | 0.34 | 1.29 | 0.09 |
| **23** | | **Nras** | 18176 | 0.25+  0.79 | 0.91+  1.54 | 1.06+  1.49 | 0.31 | 1.09 | 1.02 |
|  | | **NAME** |  | Microarray fold-change | | | qRT-PCR fold-change | | |
| **selected genes** | | |  | KNL/WNL | KTL/WNL | KTL/WTL | KNL/WNL | KTL/WNL | KTL/WTL |
| **24** | | **Kcne2** | 246133 | 0.81 | 0.29 | 0.29 | 1.01 | 0.32 | 0.25 |
| **25** | | **Ptprg** | 19270 | 0.27 | 0.37 | 0.28 | 0.57 | 0.47 | 0.31 |
| **26** | | **Lemd2** | 224640 | 0.59 | 0.99 | 1.20 | 0.29 | 0.41 | 0.47 |
| **27** | | **Pde6h** | 78600 | 2.24 | 0.89 | 1.12 | 1.25 | 0.94 | 1.11 |
| **28** | | **Olfr632** | 259123 | 3.25 | 1.23 | 0.95 | 1.32 | 0.89 | 1.15 |
| **29** | | **Emilin3** | 280635 | 2.2 | 1.19 | 1.23 | 0.89 | 1.21 | 3.21 |
| **30** | | **Larp5** | 217980 | 2.13 | 0.95 | 1.21 | 3.21 | 0.98 | 1.11 |
| **31** | | **Il12rb1** | 16161 | 2.52 | 0.85 | 0.96 | 2.24 | 1.25 | 1.32 |
| **32** | | **Hcls1** | 15163 | 2.36 | 1.95 | 0.82 | 2.59 | 0.78 | 1.01 |
| **33** | | **Tcf15** | 21407 | 2.89 | 0.86 | 1.13 | 2.21 | 0.86 | 0.76 |
| **34** | | **Npy** | 109648 | 2.48 | 1.12 | 0.97 | 1.59 | 0.89 | 0.76 |
| **35** | | **Insl6** | 27356 | 3.22 | 0.89 | 0.97 | 2.27 | 0.83 | 0.95 |
| **36** | | **Blzf1** | 66352 | 2.68 | 1.11 | 0.94 | 1.21 | 0.85 | 0.99 |
| **37** | | **Prtn3** | 19152 | 3.42 | 1.25 | 0.89 | 2.12 | 0.98 | 1.23 |
| **38** | | **Lhfp** | 108927 | 2.58 | 1.12 | 0.74 | 2.29 | 0.89 | 1.13 |
| **39** | | **Vamp1** | 22317 | 4.52 | 1.14 | 0.87 | 1.95 | 0.92 | 1.03 |
| **40** | | **Neurog1** | 18014 | 2.25 | 1.21 | 1.05 | 1.97 | 0.86 | 1.10 |
| **41** | | **Unc13d** | 70450 | 2.31 | 1.14 | 0.97 | 2.11 | 0.89 | 0.94 |
| **42** | | **Tex264** | 21767 | 3.21 | 0.89 | 1.21 | 0.68 | 2.12 | 1.31 |
| **43** | | **Acta2** | 11475 | 2.89 | 0.91 | 0.98 | 0.58 | 1.01 | 0.88 |
| **44** | | **Thoc5** | 107829 | 3.35 | 1.02 | 1.19 | 2.58 | 1.08 | 0.91 |
| **45** | | **Cd247** | 12503 | 4.85 | 1.02 | 1.32 | 2.65 | 0.98 | 1.21 |
| **46** | | **D15Wsu75e** | 28075 | 2.86 | 1.03 | 0.89 | 3.21 | 1.12 | 0.98 |
| **47** | | **6530402F18Rik** | 76220 | 3.85 | 1.25 | 1.64 | 2.28 | 1.10 | 1.08 |
| **48** | | **Ccdc109a** | 215999 | 5.26 | 1.15 | 1.21 | 3.34 | 1.01 | 0.86 |
| **49** | | **Ptpru** | 19273 | 2.26 | 1.02 | 0.78 | 1.98 | 1.13 | 0.97 |
| **50** | | **Mapk8** | 26419 | 3.31 | 0.54 | 1.25 | 2.28 | 1.24 | 2.01 |
| **51** | | **P2rx1** | 18436 | 2.59 | 0.78 | 1.25 | 2.56 | 1.54 | 1.01 |
| **52** | | **Shoc2** | 56392 | 2.89 | 0.87 | 0.98 | 3.21 | 1.01 | 1.25 |
| **53** | | **Arhgef16** | 230972 | 3.87 | 0.92 | 1.25 | 2.58 | 1.05 | 1.32 |
| **54** | | **Pdlim3** | 53318 | 4.32 | 1.10 | 1.24 | 2.25 | 1.81 | 1.36 |
| **55** | | **Gpr108** | 78308 | 3.28 | 1.02 | 1.35 | 3.58 | 0.89 | 0.97 |
| **56** | | **Klf17** | 75753 | 2.85 | 1.03 | 1.08 | 3.25 | 0.87 | 0.99 |
| **57** | | **Olfr912** | 258806 | 4.35 | 1.99 | 2.01 | 2.65 | 0.98 | 2.03 |
| **58** | | **Irf7** | 54123 | 2.58 | 1.04 | 1.29 | 3.21 | 1.89 | 2.17 |
| **59** | | **Sipa1l2** | 244668 | 2.89 | 1.21 | 1.03 | 1.86 | 0.98 | 1.45 |
| **60** | | **Tas2r119** | 57254 | 2.85 | 1.02 | 0.89 | 3.21 | 1.25 | 0.99 |
| **61** | | **Nt5c3** | 107569 | 3.58 | 1.14 | 0.99 | 2.87 | 0.86 | 1.21 |
| **62** | | **Letm2** | 270035 | 2.65 | 1.12 | 1.54 | 3.21 | 1.24 | 1.33 |
| **63** | | **Npc1l1** | 237636 | 4.21 | 1.03 | 1.54 | 2.48 | 0.88 | 0.95 |
| **64** | | **Top3a** | 21975 | 2.35 | 1.05 | 1.45 | 3.25 | 0.98 | 1.10 |
| **65** | | **Lce1a1** | 67127 | 3.33 | 1.21 | 1.05 | 3.58 | 0.89 | 0.75 |
| **66** | | **Ethe1** | 66071 | 2.24 | 1.32 | 1.07 | 2.58 | 0.87 | 0.89 |
| **67** | | **Rnf182** | 328234 | 4.21 | 0.85 | 1.06 | 3.04 | 1.05 | 1.26 |
| **68** | | **Arl5b** | 75869 | 2.58 | 1.01 | 1.25 | 3.60 | 1.00 | 1.53 |
| **69** | | **Unc5a** | 107448 | 2.89 | 1.52 | 1.28 | 0.69 | 0.87 | 0.88 |
| **70** | | **Guca1a** | 14913 | 2.58 | 1.02 | 1.22 | 3.25 | 1.21 | 1.24 |
| **71** | | **Tsga10ip** | 78306 | 2.87 | 1.32 | 1.78 | 2.21 | 1.01 | 0.98 |
| **72** | | **Gba** | 14466 | 3.54 | 1.25 | 1.02 | 2.58 | 1.21 | 1.08 |
| **73** | | **Vwa2** | 240675 | 4.23 | 1.27 | 1.03 | 2.21 | 1.01 | 1.15 |
| **74** | | **Slc24a6** | 170756 | 2.58 | 1.27 | 1.41 | 2.65 | 0.89 | 1.77 |
| **75** | | **Me2** | 107029 | 2.54 | 1.02 | 1.11 | 3.58 | 2.01 | 1.25 |
| **76** | | **Nr1d2** | 353187 | 3.62 | 0.82 | 0.98 | 4.25 | 1.58 | 1.45 |
| **77** | | **Asphd1** | 233879 | 2.54 | 0.87 | 0.88 | 2.25 | 1.03 | 1.52 |
| **78** | | **Ensa** | 56205 | 2.98 | 0.85 | 1.11 | 4.56 | 1.89 | 1.21 |
| **79** | | **Olfr458** | 258436 | 2.89 | 1.27 | 2.21 | 0.59 | 2.01 | 1.06 |
| **80** | | **Spred2** | 114716 | 3.21 | 1.11 | 0.89 | 2.58 | 1.23 | 1.54 |
| **81** | | **Acot5** | 217695 | 2.25 | 1.32 | 1.25 | 3.40 | 1.26 | 2.21 |
| **82** | | **Clec7a** | 56644 | 4.54 | 1.02 | 0.82 | 3.54 | 1.25 | 1.21 |
| **83** | | **Stxbp4** | 20913 | 2.65 | 0.78 | 1.21 | 3.54 | 1.04 | 1.77 |
| **84** | | **Hnf4g** | 30942 | 3.56 | 1.85 | 1.20 | 3.25 | 1.28 | 1.89 |
| **85** | | **Nubp2** | 26426 | 2.89 | 1.28 | 2.21 | 3.58 | 1.12 | 1.89 |
| **86** | | **Rassf1** | 56289 | 4.56 | 1.21 | 1.00 | 3.25 | 0.85 | 0.99 |
| **87** | | **Drd1ip** | 68566 | 2.65 | 1.21 | 1.11 | 3.56 | 1.21 | 1.01 |
| **88** | | **Snrpa** | 53607 | 3.57 | 0.78 | 1.10 | 4.21 | 0.98 | 1.11 |
| **89** | | **Gapdhs** | 14447 | 2.25 | 1.01 | 1.87 | 4.35 | 1.03 | 1.10 |
| **90** | | **Galnt1** | 14423 | 3.28 | 1.05 | 1.01 | 3.58 | 1.02 | 2.03 |
| **91** | | **Vamp8** | 22320 | 2.55 | 0.89 | 0.78 | 4.06 | 0.87 | 1.02 |
| **92** | | **Top3b** | 21976 | 3.58 | 0.89 | 1.02 | 2.02 | 1.08 | 1.87 |
| **93** | | **Cdc34** | 216150 | 2.21 | 0.98 | 1.12 | 0.58 | 0.67 | 1.15 |
| **94** | | **Gpc6** | 23888 | 2.54 | 1.21 | 1.10 | 3.25 | 1.02 | 0.98 |
| **95** | | **Gdf10** | 14560 | 2.58 | 1.05 | 1.11 | 0.69 | 0.84 | 0.54 |
| **96** | | **Pold4** | 69745 | 3.58 | 1.21 | 1.10 | 2.88 | 1.20 | 1.15 |
| **97** | | **Pramel7** | 347712 | 4.28 | 2.05 | 1.12 | 1.89 | 1.77 | 1.21 |
| **98** | | **Bmp2** | 12156 | 2.35 | 1.02 | 1.11 | 3.57 | 1.41 | 0.89 |
| **99** | | **Pmf1** | 67037 | 2.69 | 1.19 | 0.87 | 3.02 | 1.21 | 1.15 |
| **100** | | **Aqp6** | 11831 | 3.41 | 1.25 | 1.54 | 2.38 | 0.89 | 0.85 |
