# Supplementary material for: Behavior Training Reverses Asymmetry in Hippocampal Transcriptome of the Cav3.2 Knockout Mice
Source: PLoS One. 2015 Mar 13;10(3):e0118832. doi: 10.1371/journal.pone.0118832 (PMC4358833; doi:10.1371/journal.pone.0118832)
Supplement: S3 Table — (DOCX) [file pone.0118832.s005.docx]

| **Table S3: The enriched pathways with 10 or more DEGs in KNL vs WNL comparison** | | |
| --- | --- | --- |
| 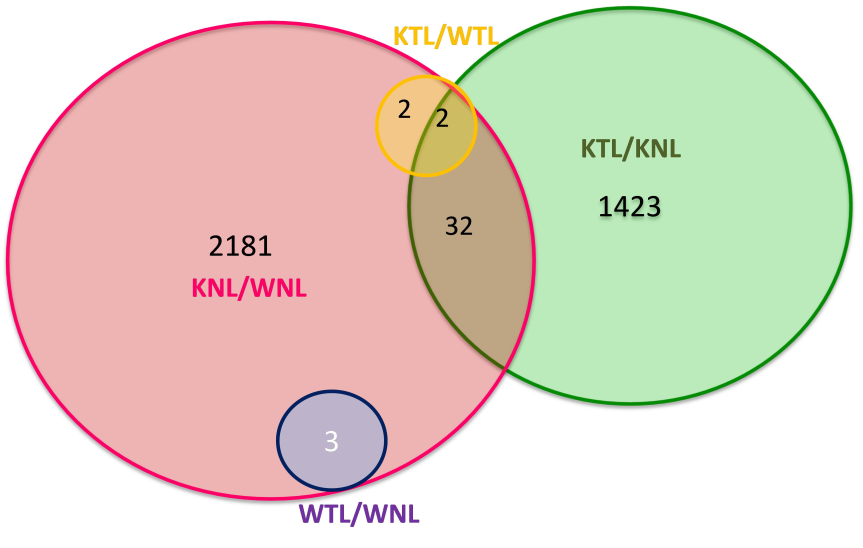**KEGG pathway**  UP-Regulated Gene Numbers | **Count** | **Benjamini adjusted p-value** |
| **KNL vs WNL** |  |  |
| mmu04010:MAPK signaling pathway | 45 | 4.69E-04 |
| mmu05200:Pathways in cancer | 42 | 6.53E-02 |
| mmu04120:Ubiquitin mediated proteolysis | 38 | 7.64E-09 |
| mmu04144:Endocytosis | 35 | 1.61E-03 |
| mmu05010:Alzheimer's disease | 30 | 7.64E-03 |
| mmu04810:Regulation of actin cytoskeleton | 30 | 6.51E-02 |
| mmu05016:Huntington's disease | 29 | 1.48E-02 |
| mmu03040:Spliceosome | 26 | 4.73E-04 |
| mmu04722:Neurotrophin signaling pathway | 25 | 2.20E-03 |
| mmu04310:Wnt signaling pathway | 24 | 2.33E-02 |
| mmu00190:Oxidative phosphorylation | 22 | 1.85E-02 |
| mmu04114:Oocyte meiosis | 21 | 9.78E-03 |
| mmu05012:Parkinson's disease | 21 | 4.19E-02 |
| mmu04530:Tight junction | 21 | 4.79E-02 |
| mmu05414:Dilated cardiomyopathy | 17 | 1.96E-02 |
| mmu04912:GnRH signaling pathway | 16 | 5.85E-02 |
| mmu03018:RNA degradation | 14 | 5.61E-03 |
| mmu04720:Long-term potentiation | 14 | 2.04E-02 |
| mmu05220:Chronic myeloid leukemia | 14 | 3.77E-02 |
| mmu05210:Colorectal cancer | 14 | 8.62E-02 |
| mmu00020:Citrate cycle (TCA cycle) | 13 | 2.20E-05 |
| mmu03050:Proteasome | 13 | 1.81E-03 |
| mmu05412:Arrhythmogenic right ventricular cardiomyopathy (ARVC) | 13 | 6.88E-02 |
| mmu04260:Cardiac muscle contraction | 13 | 8.72E-02 |
| mmu05211:Renal cell carcinoma | 12 | 8.78E-02 |
| mmu00280:Valine, leucine and isoleucine degradation | 11 | 1.40E-02 |
| mmu04150:mTOR signaling pathway | 11 | 3.99E-02 |
| mmu04621:NOD-like receptor signaling pathway | 11 | 8.77E-02 |
| mmu00071:Fatty acid metabolism | 10 | 3.21E-02 |
| mmu04130:SNARE interactions in vesicular transport | 9 | 3.20E-02 |
| mmu03420:Nucleotide excision repair | 9 | 6.11E-02 |
| mmu00601:Glycosphingolipid biosynthesis | 6 | 8.58E-02 |
| mmu00630:Glyoxylate and dicarboxylate metabolism | 5 | 6.89E-02 |
| mmu00100:Steroid biosynthesis | 5 | 8.33E-02 |
| **KTL vs WTL** |  |  |
| mmu04740:Olfactory transduction | 83 | 4.04E-02 |
| mmu05200:Pathways in cancer | 31 | 8.04E-02 |
| mmu04080:Neuroactive ligand-receptor interaction | 28 | 3.00E-02 |
| mmu04060:Cytokine-cytokine receptor interaction | 27 | 2.49E-02 |
| mmu04010:MAPK signaling pathway | 27 | 5.92E-02 |
| mmu04630:Jak-STAT signaling pathway | 23 | 1.06E-03 |
| mmu04640:Hematopoietic cell lineage | 16 | 7.64E-04 |
| mmu04660:T cell receptor signaling pathway | 14 | 7.70E-02 |
| mmu05222:Small cell lung cancer | 13 | 1.69E-02 |
| mmu05215:Prostate cancer | 13 | 2.55E-02 |
| mmu05217:Basal cell carcinoma | 12 | 1.47E-03 |
| mmu00590:Arachidonic acid metabolism | 12 | 3.30E-02 |
| mmu04916:Melanogenesis | 12 | 9.92E-02 |
| mmu00830:Retinol metabolism | 10 | 5.13E-02 |
| mmu05223:Non-small cell lung cancer | 9 | 3.63E-02 |
| mmu05340:Primary immunodeficiency | 8 | 1.23E-02 |
| mmu04340:Hedgehog signaling pathway | 8 | 8.77E-02 |
| mmu00531:Glycosaminoglycan degradation | 5 | 7.72E-02 |


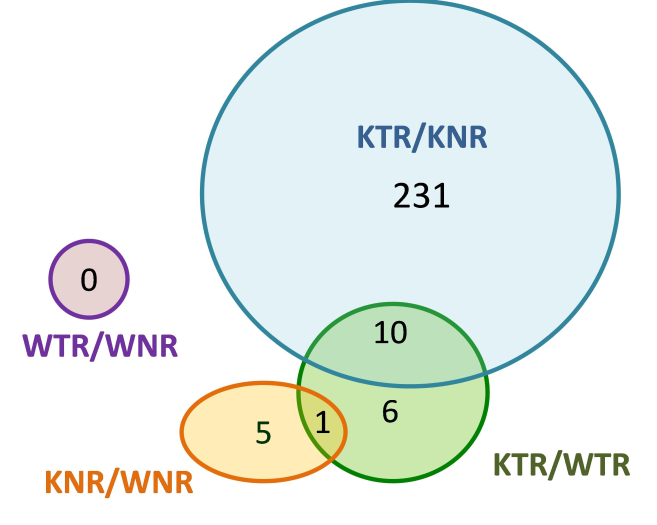


| **KEGG pathway** | **Count** | **Benjamini adjusted p-value** |  |
| --- | --- | --- | --- |
| **KTR vs KNR** |  |  |  |
| mmu03040:Spliceosome | 6 | 2.37E-02 |  |
| mmu05010:Alzheimer's disease | 6 | 9.26E-02 |  |
| mmu05016:Huntington's disease | 6 | 9.43E-02 |  |
| mmu00190:Oxidative phosphorylation | 5 | 9.29E-02 |  |
| **KNR vs WNR** |  |  |  |
| mmu04120:Ubiquitin mediated proteolysis | 1 |  |  |
| **KTR vs WTR** |  |  |  |
| mmu00190:Oxidative phosphorylation | 1 |  |  |
| mmu05010:Alzheimer's disease | 1 |  |  |
| mmu05012:Parkinson's disease | 1 |  |  |
| mmu05016:Huntington's disease | 1 |  |  |
| mmu04144:Endocytosis | 1 |  |  |
| mmu04080:Neuroactive ligand-receptor interaction | 1 |  |  |
|  |  |  |  |
| mmu04910:Insulin signaling pathway | 1 |  |  |


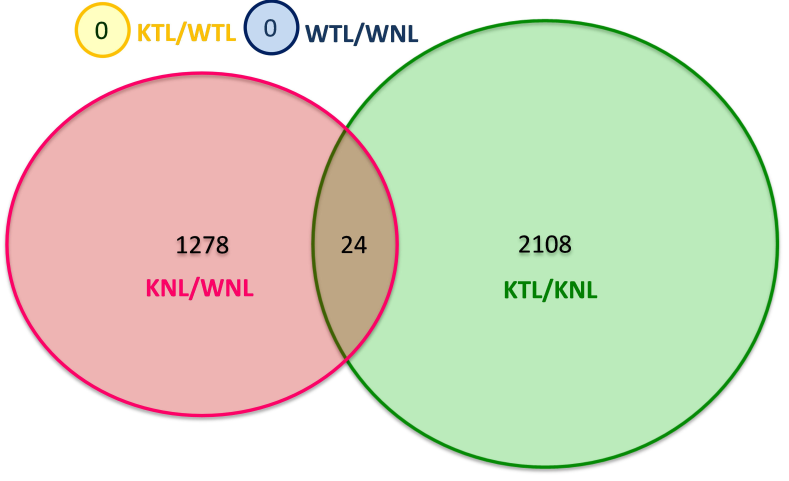


Down-regulated gene numbers

| **KEGG pathway** | **Count** | **Benjamini adjusted p-value** |
| --- | --- | --- |
| **KNL vs WNL** |  |  |
| mmu04010:MAPK signaling pathway | 28 | 1.34E-02 |
| mmu04310:Wnt signaling pathway | 19 | 7.99E-03 |
| mmu05217:Basal cell carcinoma | 13 | 1.71E-04 |
| mmu04670:Leukocyte transendothelial migration | 13 | 8.71E-02 |
| mmu04640:Hematopoietic cell lineage | 12 | 2.00E-02 |
| mmu05222:Small cell lung cancer | 11 | 4.92E-02 |
| mmu00830:Retinol metabolism | 10 | 3.16E-02 |
| mmu00982:Drug metabolism | 10 | 5.43E-02 |
| mmu00590:Arachidonic acid metabolism | 10 | 9.06E-02 |
| mmu04920:Adipocytokine signaling pathway | 9 | 6.90E-02 |
| mmu04340:Hedgehog signaling pathway | 8 | 6.01E-02 |
| mmu05216:Thyroid cancer | 6 | 3.76E-02 |
| mmu00531:Glycosaminoglycan degradation | 5 | 5.92E-02 |
| **KTL vs WTL** |  |  |
| mmu04010:MAPK signaling pathway | 41 | 4.25E-03 |
| mmu04120:Ubiquitin mediated proteolysis | 38 | 5.72E-09 |
| mmu04144:Endocytosis | 34 | 2.60E-03 |
| mmu05010:Alzheimer's disease | 27 | 3.56E-02 |
| mmu04310:Wnt signaling pathway | 26 | 5.83E-03 |
| mmu05016:Huntington's disease | 26 | 6.08E-02 |
| mmu03040:Spliceosome | 23 | 4.80E-03 |
| mmu04722:Neurotrophin signaling pathway | 21 | 3.08E-02 |
| mmu00190:Oxidative phosphorylation | 21 | 3.08E-02 |
| mmu04530:Tight junction | 21 | 4.37E-02 |
| mmu05012:Parkinson's disease | 20 | 6.57E-02 |
| mmu04114:Oocyte meiosis | 19 | 3.37E-02 |
| mmu05414:Dilated cardiomyopathy | 17 | 1.79E-02 |
| mmu04666:Fc gamma R-mediated phagocytosis | 17 | 3.09E-02 |
| mmu03050:Proteasome | 15 | 1.19E-04 |
| mmu05412:Arrhythmogenic right ventricular cardiomyopathy (ARVC) | 14 | 3.18E-02 |
| mmu05220:Chronic myeloid leukemia | 14 | 3.50E-02 |
| mmu03018:RNA degradation | 13 | 1.34E-02 |
| mmu04720:Long-term potentiation | 13 | 4.12E-02 |
| mmu00020:Citrate cycle (TCA cycle) | 12 | 1.12E-04 |
| mmu00280:Valine, leucine and isoleucine degradation | 12 | 4.44E-03 |
| mmu04150:mTOR signaling pathway | 12 | 1.54E-02 |
| mmu05211:Renal cell carcinoma | 12 | 8.29E-02 |
| mmu00071:Fatty acid metabolism | 11 | 1.12E-02 |
| mmu04130:SNARE interactions in vesicular transport | 10 | 1.04E-02 |
| mmu03420:Nucleotide excision repair | 9 | 5.81E-02 |
| mmu00100:Steroid biosynthesis | 8 | 7.35E-04 |
| mmu00640:Propanoate metabolism | 8 | 2.47E-02 |


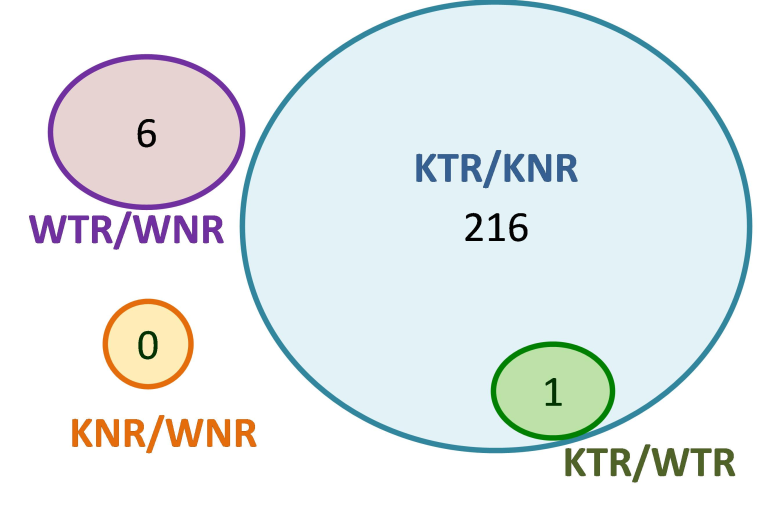


| **KEGG pathway** | **Count** | **Benjamini adjusted p-value** |
| --- | --- | --- |
| **KTR vs KNR** |  |  |
| mmu00830:Retinol metabolism | 5 | 3.66E-03 |
| mmu00980:Metabolism of xenobiotics by cytochrome P450 | 4 | 2.38E-02 |
| mmu00982:Drug metabolism | 4 | 3.31E-02 |
| mmu05217:Basal cell carcinoma | 3 | 9.41E-02 |
